# Deformation of a Capsule in a Power-Law Shear Flow

**DOI:** 10.1155/2016/7981386

**Published:** 2016-10-19

**Authors:** Fang-Bao Tian

**Affiliations:** School of Engineering and Information Technology, University of New South Wales, Canberra, ACT 2600, Australia

## Abstract

An immersed boundary-lattice Boltzmann method is developed for fluid-structure interactions involving non-Newtonian fluids (e.g., power-law fluid). In this method, the flexible structure (e.g., capsule) dynamics and the fluid dynamics are coupled by using the immersed boundary method. The incompressible viscous power-law fluid motion is obtained by solving the lattice Boltzmann equation. The non-Newtonian rheology is achieved by using a shear rate-dependant relaxation time in the lattice Boltzmann method. The non-Newtonian flow solver is then validated by considering a power-law flow in a straight channel which is one of the benchmark problems to validate an in-house solver. The numerical results present a good agreement with the analytical solutions for various values of power-law index. Finally, we apply this method to study the deformation of a capsule in a power-law shear flow by varying the Reynolds number from 0.025 to 0.1, dimensionless shear rate from 0.004 to 0.1, and power-law index from 0.2 to 1.8. It is found that the deformation of the capsule increases with the power-law index for different Reynolds numbers and nondimensional shear rates. In addition, the Reynolds number does not have significant effect on the capsule deformation in the flow regime considered. Moreover, the power-law index effect is stronger for larger dimensionless shear rate compared to smaller values.

## 1. Introduction

Flow induced deformation of a capsule consisting of a membrane enclosing an internal medium such as a gel or a liquid is an important problem in fundamental research as well as bioengineering applications. For example, a capsule in shear flow is a fundamental process that is related to erythrocytes (or red blood cells), leukocytes (or white blood cells), and platelets in blood flow [1–6]. Deformation is essential for red blood cells to perform their physiological functions in the circulation of capillary blood vessels and thus affects the rheology of the blood [6–8]. The deformations of white blood cells and red blood cells can, respectively, affect the immune response and the oxygen load release [9, 10]. The synthetic microcapsules with polymerized interfaces are designed for drug delivery, cosmetic production, and other technical usages [11, 12]. Therefore, great effort has been made to study this problem (e.g., [1, 4, 6, 8, 10, 12–14]).

Both experimental and numerical methods have been conducted to observe capsule behaviors and the relevant underneath fluid-structure interaction physics. Schmid-Schönbein and Wells [15] and Goldsmith [16] observed that red blood cells tumble like rigid particles at low shear rates while they deform to a steady configuration and direction after which the membrane rotates around the internal liquid (tank-treading movement) at high shear rates. Later, Goldsmith and Marlow [17] and Keller and Skalak [18] found that the viscosity ratio between the liquids inside and outside the cell may also affect the type of behaviors. A higher viscosity inside would cause unsteady tumbling-rotating motion, while a smaller viscosity inside would lead to the tank-treading movement with a stationary shape. These phenomena were captured by Xu et al. [14]. More recently, Dupire et al. [19] reported rolling motion in addition to other behaviors. A hysteresis cycle and two transient dynamics driven by the shear rate (i.e., an intermittent regime during the “tank-treading-to-flipping” transition and a Frisbee-like “spinning” regime during the “rolling-to-tank-treading” transition) were highlighted.

There are several numerical methods that have been used to study capsule dynamics. Examples are the boundary element method (e.g., [20]), arbitrary Lagrangian-Euler method (e.g., [21–23]), immersed finite element method (e.g., [24]), and immersed boundary method (IBM) (e.g., [12–14, 25–34]). Specifically, Zhou and Pozrikidis [20] studied the transient and large deformation of capsules with position-dependent membrane tension. Choi and Kim [21] simulated the motion of red blood cells freely suspended in shear flow to investigate the nature of pairwise interception of red blood cells using a fluid-particle interaction method based on the arbitrary Lagrangian–Eulerian method. Villone et al. [22, 23] studied the effect of the non-Newtonian fluid on flexible particle deformation and migration in shear and channel flows by using the arbitrary Lagrangian–Eulerian method. The Navier–Stokes equations and cell-cell interaction were coupled in the framework of the immersed finite element method and mesh-free method by Y. Liu and W. K. Liu [24] to model complex blood flows with deformable red blood cells within micro and capillary vessels in three dimensions. The transient deformation of a liquid-filled elastic capsule in simple shear flow was studied by Sui et al. [1, 4, 35, 36]. The fluid inertia on the dynamics of deformable particles has been studied by Krüger et al. [32] and Kaoui and Harting [34]. More recently, optical force based separation of particles/capsules was simulated by Chang et al. [37–39]. Still, as far as known to us, the existing numerical simulations seldom consider the non-Newtonian rheology effects on the capsule behaviors, while blood and most fluids involved in biomedical engineering are non-Newtonian fluids [6, 8, 40, 41].

Following the work by Sui et al. [1] and Xu et al. [14], we develop an immersed boundary-lattice Boltzmann method (IB-LBM) to study the non-Newtonian effects on the deformation of a capsule in a shear flow. As a typical rheology, the power-law fluid is used. In this method, the capsule dynamics and the fluid dynamics are coupled by using the IBM, and the incompressible viscous power-law fluid motion is acquired by solving the lattice Boltzmann equation (LBE).

The rest of this paper is organized as follows.  Section 2 briefly introduces the governing equations of the fluid and solid structures and describes the numerical approach.  Section 3 presents the numerical results. Final conclusions are given in Section 4.

## 2. Mathematical Formulation and Numerical Method

### 2.1. Physical Model and Mathematical Formulation

**Figure 1:**
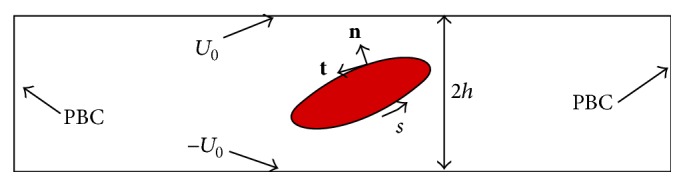
Schematic illustration of a circular liquid capsule immersed in a fluid.

In this work, a two-dimensional liquid capsule enclosed by an elastic membrane and immersed in an incompressible non-Newtonian fluid is considered, as illustrated in Figure 1 where *s* is the arch length coordinate, **n** denotes the surface normal that points into the outer fluid, **t** denotes the tangent unit vector that points to the increasing arc length, and *U*_0_ is the velocity applied at both top and bottom walls to form a simple shear flow. The incompressible non-Newtonian fluid dynamics is achieved by using LBM [42, 43]. Great effort has been made in using LBM to solve the complex flows (see several reviews [42–44] for the effort). Many publications have presented the details of LBM; thus we just provide a brief description in this paper and discuss the extension for non-Newtonian fluids. The details of LBM and its applications are referred to the references provided. Using the IB-LBM, the lattice Boltzmann equation (LBE) that governs the viscous flow dynamics and incorporates the traction jump across the interface due to the elastic membrane is written as [1, 14, 42, 43, 45, 46]

(1) gix+eiΔt,t+Δt−gix,t=−1τLBgix,t−gieqx,t+ΔtGi,

(2) gieq=ωiρ1+ei·ucs2+uu:eiei−cs2I2cs4,

(3) Gi=1−12τLBωiei−ucs2+ei·ucs4ei·f,

(4) fx,t=∫ΔFs,tδDx−Xs,tds,

where *g*_*i*_(**x**, *t*) is the distribution function for particles with velocity **e**_*i*_ at position **x** and time *t*, Δ*t* is the size of the time step, *g*_*i*_^eq^(**x**, **t**) is the equilibrium distribution function, *τ*_LB_ represents the dimensionless relaxation time, *G*_*i*_ is the term representing the body force effect on the distribution function, *ω*_*i*_ are the weights, **u** = (*u*, *v*) is the velocity of the fluid, *c*_*s*_ is the speed of sound defined by $c_{s}=\Delta x/\sqrt{3}\Delta t$ with Δ*x* being grid spacing, **f** is the body force acting on the fluid, Δ**F**(*s*, *t*) is the Lagrangian force density on the fluid by the elastic boundary, **X** is the position vector on the membrane, and *δ*_*D*_(**x** − **X**(**s**, *t*)) is Dirac's delta function.

**Figure 2:**
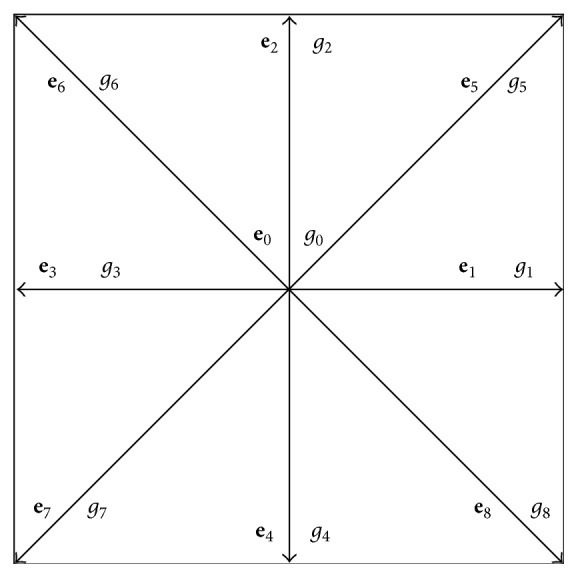
Nine base vectors representing 9 possible velocity directions in the D2Q9 lattice model.

In the present work, a two-dimensional nine-speed (D2Q9) model is used, as shown in Figure 2. In this model, the nine possible particle velocities are given by

(5) e0=0,0,ei=cos⁡πi−12,sin⁡πi−12ΔxΔt,for  i=1  to  4,ei=cos⁡πi−9/22,sin⁡πi−9/222ΔxΔt,for  i=5  to  8.

The values of **e**_*i*_ ensure that, within one time step, a particle moves to one of the eight neighboring nodes as shown in Figure 2 or stays at its current location. The weights, *ω*_*i*_, are given by *ω*_0_ = 4/9 and *ω*_*i*_ = 1/9 for *i* = 1 to 4 and *ω*_*i*_ = 1/36 for *i* = 5 to 8. In addition, the relaxation time is related to the kinematic viscosity in the Navier–Stokes equations in terms of

(6) $$\begin{array}{c} \tau_{\text{LB}}=0.5+\frac{\nu }{c_{s}^{2}\Delta t}, \end{array}$$

where *ν* = *μ*/*ρ* with *μ* being the dynamic viscosity of the ambient fluid and *ρ* being the fluid density.

When the particle density distributions are known, the fluid density, velocity, and pressure are then computed from

(7) ρ=∑igi,u=∑ieigi+0.5fΔtρ,p=ρcs2.

Theoretically the LBM introduced above simulates the compressible viscous flow instead of incompressible viscous one, because the spatial density variation is not zero in LBM simulations. In the applications, the Mach number (Ma = *u*_0_/*c*_*s*_) should be low (e.g., Ma ≤ 0.3) so that the incompressible viscous flow can be correctly simulated. The deduction process from LBE to the incompressible viscous flow governing equations can be found in [47].

The dynamics viscosity is a constant for a Newtonian fluid, while it is dependent on the local shear rate for a non-Newtonian fluid. Without loss of generality, the power-law fluid is taken as a representation of non-Newtonian fluids in the present paper. The rheological equation of state for power-law fluids is defined by [48]

(8) μ=ηγ˙n−1,

(9) γ˙=max⁡2EijEij,γ˙m,

(10) Eij=12∂ui∂xj+∂uj∂xi,

where *η* is the power-law consistency index, *n* is the power-law fluid behavior index, $\dot{\gamma }$ is the shear rate, and ${\dot{\gamma }}_{m}$ is the minimum shear rate that is applied to avoid the numerical singularity caused by the zero shear rate. The power-law fluids of *n* < 1, *n* > 1 and *n* = 1 are, respectively, the shear-thinning, shear-thickening, and Newtonian fluids. In (9), the Einstein summation convention is applied. In LBM implementation, *E*_*ij*_ can be either calculated macroscopically by using the finite difference method or locally in mesoscopic scale by using *g* ^eq^ and **f** [49]. To achieve the non-Newtonian rheology, a shear rate-dependant relaxation time is used which can be obtained by applying the effective viscosity determined by (8) in (6).

Because of the deformation, the membrane develops a transverse shear tension *q* and a bending moment *m*. In addition, due to the stretching motion, a tension, *τ*, is induced. Consider the force balance of membranes; we acquire

(11) ΔF=∂∂sqn+τt,q=∂m∂s.

Please note that Δ**F** is the Lagrangian force on the fluid exerted by the elastic body boundary and is opposite to the fluid force on the boundary. To evaluate *m* and *τ* for the thin membrane, we use Hooke's law which is a relatively simpler constitutive law for modeling small deformation of capsules. Hooke's law states that the tension and the bending moment are linearly related to the stretch and the curvature, respectively. It can be written in the form

(12) m=EBκ−κ0,τ=ES∂X∂s0−1,

where *E*_*B*_ is the bending coefficient, *E*_*S*_ is the stretching coefficient, *s*_0_ is the initial arch length, *κ* is the curvature of membrane, and *κ*_0_ is the curvature in the resting configuration. If *E*_*S*_ is large so that the stretching deformation is small, Hooke's law works well. Following the work by Sui et al. [1], the capsule membrane is assumed to be infinitely thin so that the bending effect is neglected; that is, *E*_*B*_ = 0. Actually, the effect of *E*_*B*_ is similar to that of *E*_*S*_ when *E*_*B*_ is small compared to *E*_*S*_ [1, 35]. If *E*_*B*_ is large, the capsule may undergo flipping motion [35].

The velocity of a point on the capsule is interpolated from the flow field, and the position of the capsule is updated explicitly; that is,

(13) Us,t=∫ux,tδDx−Xs,tdx,

(14) ∂Xs,t∂t=Us,t,

where **U**(*s*, *t*) is the velocity of the capsule.

In this work, we choose the flow shear rate (e.g., *U*_0_/*h*), density, and the radius of the capsule to nondimension the governing equations and obtain two dimensionless parameters: the Reynolds number Re and dimensionless shear rate *G*, which are defined by

(15) Re=ρU2−nLnη=ρ2a2ηU0h2−n,

(16) G=ηaESU0hn,

where *a* is the radius of the undeformed capsule. *G* measures the ratio of shear force to the elastic force. For applications where inertia force is important, we can also use Re · *G* to nondimension the elastic property, which measures the ratio of fluid inertial forces to stretching elastic forces. Please note that the two-dimensional model is used in this work, while red blood cell deformation is a three-dimensional problem; however, the results obtained in this research should show some features common with the three-dimensional simulations, as demonstrated in [1].

### 2.2. Numerical Method

Similar to [1], the capsule is discretized by *N*_*f*_ nodal points which are initially distributed with equal distances. The position of the *m*th node at time level *n* is denoted by **X**_*m*_^*n*^. To compute the stretching force at *m*th node, a finite difference scheme is used; that is,

(17) $$\begin{array}{c} \frac{\partial }{\partial s}{\left[\;\tau \left(\;s\;\right)\frac{\partial X}{\partial s}\;\right]}_{m}=\frac{\tau_{m+1/2}t_{m+1/2}-\tau_{m-1/2}t_{m-1/2}}{\Delta s}, \end{array}$$

where Δ*s* is the Lagrangian grid spacing on the membrane and the tension *τ* and tangent vector, **t** = ∂**X**/∂*s*, at the segment center, *m* + 1/2, are both computed using a second-order central difference scheme.

The time integration of (14) is calculated according to

(18) $$\begin{array}{c} X^{n+1}=X^{n}+\Delta tU^{n+1}. \end{array}$$

In the IBM, a smooth approximation [50] of Dirac's delta function, *δ*_*h*_, is used,

(19) δhx=1ΔxΔyϕxΔxϕyΔy,ϕr=1+cos⁡πr/24r<2,0,r≥2.

In the present simulations, Δ*x* = Δ*y* = Δ*t* (in lattice units) is used.

Now, the computational algorithm can be summarized as follows. Given all values at time step *n*, the values at time step *n* + 1 can be undated by the following:

(1) Calculate the Lagrangian force density Δ**F** ^*n*+1^ from **X** ^*n*^ by using (11)-(12).
(2) Spread the Lagrangian force density Δ**F** ^*n*+1^ onto the ambient fluid nodes by using (4), and obtain **f** ^*n*+1^.
(3) Solve flow field with body force by using the LBM method described by (1)–(3) and (6)–(10).
(4) Update **U** ^*n*+1^ by using (13).
(5) And finally, update **X** ^*n*+1^ by using (18).

In the present work, the above-mentioned computational simulation algorithm is implemented in the Fortran 90 programming language.

### 2.3. Validation

**Figure 3:**
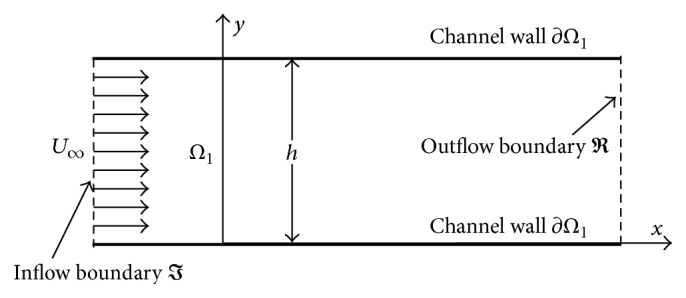
Sketch of the power-law fluid flow in a channel.

The IB-LBM in this work has been validated and verified in our previous studies (see, e.g., [14, 46]) and has been used to study filament(s) flapping in viscous fluids [51–53], sperm swimming, and cell/particle flows [10, 54]. In the present work, we focus on the validation of non-Newtonian flow by considering a power-law flow in a straight channel which is one of the benchmark problems to validate an in-house computational fluid dynamics solver. As in our previous work [41], we consider a two-dimensional steady laminar developing flow of power-law fluid with a uniform incoming velocity *U*_*∞*_ through a rectangular channel of height *h* and length *L*, as shown in Figure 3. The physically realistic initial and boundary conditions are given as

(20) ux,y=0,vx,y=0,px,y=0,t=0,  x,y∈Ω1,

(21) ux,y=U∞,vx,y=0,t>0,  x,y∈I,vx,y=0,t>0,  x,y∈∂Ω1,t>0,  x,y∈R.

(22) ux,y=0,vx,y=0,t>0,  x,y∈∂Ω1,t>0,  x,y∈R.

(23) px,y=p0,t>0,  x,y∈R.

**Figure 4:**
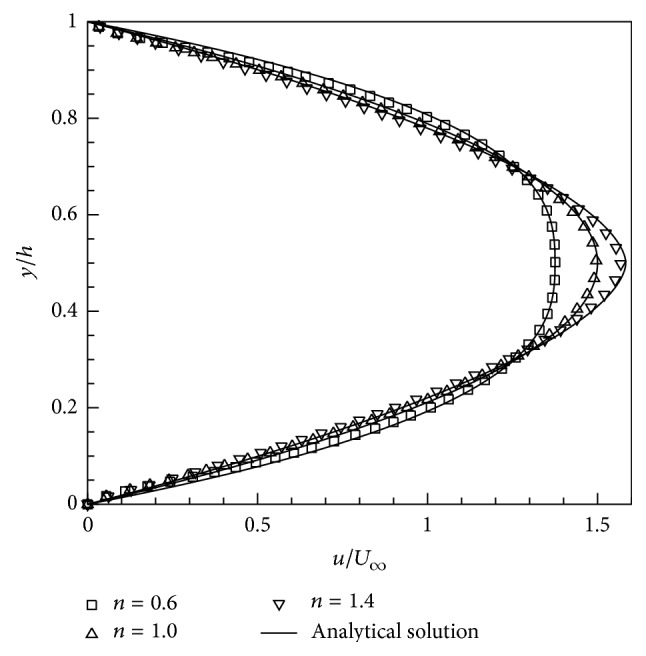
Comparison of the present numerical results of the fully developed velocity profiles in a channel with the corresponding analytical profiles (24) for Reynolds number of Re = 100 and power-law indices *n* = 0.6, 1.0, and 1.4.

The computations are performed with the dimensionless domain size (*L*/*h*  ×  1) of 40 × 1 discretized by 2001 × 51 uniform Cartesian nodes. The numerical results in terms of the fully developed velocity profiles are obtained for the Reynolds number (defined by *ρh* ^*n*^ *U*_*∞*_^2−*n*^/*η*) of 100 and for three power-law indices; that is, *n* = 0.6, 1.0, and 1.4. The simulations are performed for sufficiently long time so that the flow in the channel attains a steady state. The fully developed velocity profiles predicted by the numerical simulations are compared in Figure 4 with the corresponding analytical solution for fully developed velocity profile [41, 48] for power-law fluid flow in a channel which is given as

(24) $$\begin{array}{c} \frac{u\left(y,n\right)}{U_{\infty }}=\frac{2n+1}{n+1}\left(\;1-{\left|\;1-\frac{2y}{h}\;\right|}^{\left(n+1\right)/n}\;\right). \end{array}$$

From Figure 4, it is found that the present numerical results show a good agreement with the analytical solutions for various values of power-law index, giving us confidence in the reliability and accuracy of the present numerical solution procedure. It is noted from Figure 4 that the shear layer is thinned for *n* < 1 and thickened for *n* > 1 compared to the Newtonian fluid case (*n* = 1).

## 3. Numerical Results

We first consider the power-law index effect on the deformation of a cylindrical capsule in a shear flow. The Reynolds number is 0.05, which is in the range of normal physiological conditions. The dimensionless shear rate *G* is 0.04. The computational domain ranges from 0 to 20*a* in both *x*-axis and *y*-axis. The capsule is at the center of the domain, and its membrane is equally discretized into 80 Lagrangian nodes. The grid resolution is Δ*x* = Δ*y* = Δ*t* = 0.1*a*. The characteristic velocity is set as *U*_0_ = 0.05 so that the dimensionless relaxation time is 0.5 < *τ*_LB_ < 3.0. Such setup is consistent with that used in [1]. To study the power-law index effect, *n* is set in the range of 0.2 < *n* < 1.8, covering the shear-thinning, Newtonian, and shear-thickening fluids. In order to quantify the deformation of the capsule, the Taylor shape parameter *D*_*xy*_ is introduced [1],

(25) $$\begin{array}{c} D_{xy}=\frac{L-B}{L+B}, \end{array}$$

where *L* and B are, respectively, the length and width of a cross-section of the cylindrical capsule.

**Figure 5:**
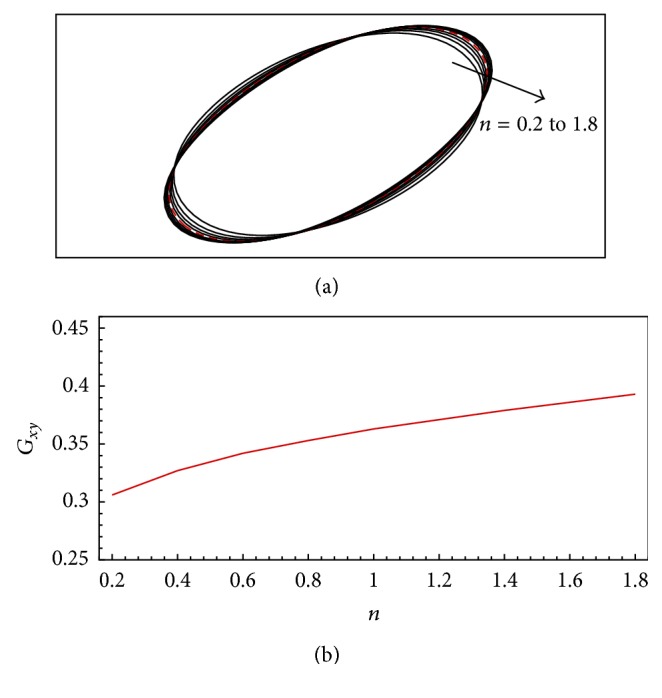
Deformation of the flexible capsule in a shear flow for Reynolds number of Re = 0.05, dimensionless shear rate of *G* = 0.04, and power-law index of *n* = 0.2 to 1.8: (a) capsule shapes for difference power-law indices (the dashed line is for the Newtonian fluid case where *n* = 1.0) and (b) Taylor shape parameter as a function of power-law index.

**Figure 6:**
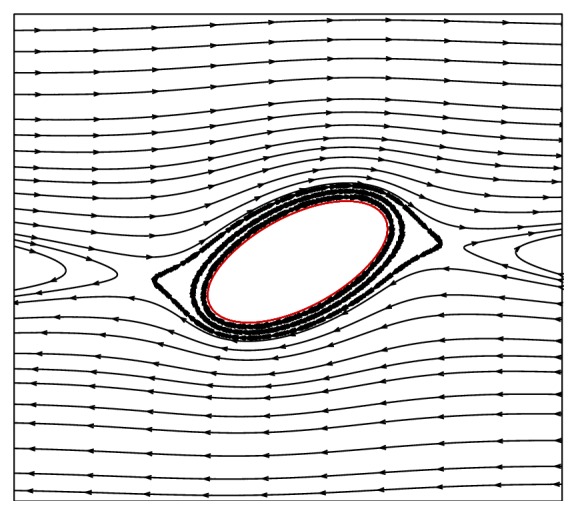
The streamline pattern inside and outside the capsule at steady state for Re = 0.05, *G* = 0.04, and *n* = 1.0.

Figure 5 shows the deformation of the flexible capsule in a shear flow for Reynolds number of Re = 0.05, dimensionless shear rate of *G* = 0.04, and power-law index of *n* = 0.2 to 1.8. There are several interesting observations from Figure 5. First, the capsule deforms to a steady shape and then the membrane rotates around the liquid inside (tank-treading motion), which is further indicated by the streamlines in Figure 6. Second, the deformation increases with the power-law index. When the fluid is shear-thinning (i.e., *n* < 1.0), the deformation is smaller compared to the Newtonian fluid case (*n* = 1.0), while the deformation is larger compared to the Newtonian fluid case for the shear-thickening fluid; that is, *n* > 1.0. This can be explained by the power-law rheology. When *n* < 1.0, the effective viscosity near the capsule is smaller compared to the Newtonian fluid, while the effective viscosity near the capsule is higher than that of Newtonian fluid for *n* > 1.0. Based on the definition of *G* in (16), the local *G* is larger for *n* > 1.0 and smaller for *n* < 1.0 compared to that of Newtonian fluid. As presented by Sui et al. [1], a larger *G* corresponds to a larger *G*_*xy*_, that is, larger deformation of the capsule. Third, the Taylor shape parameter *G*_*xy*_, which is used to quantify the deformation, increases with the power-law index. Finally, it is noted that *G*_*x*y_ is approximately linear function of *n*, as shown in Figure 5(b).

**Figure 7:**
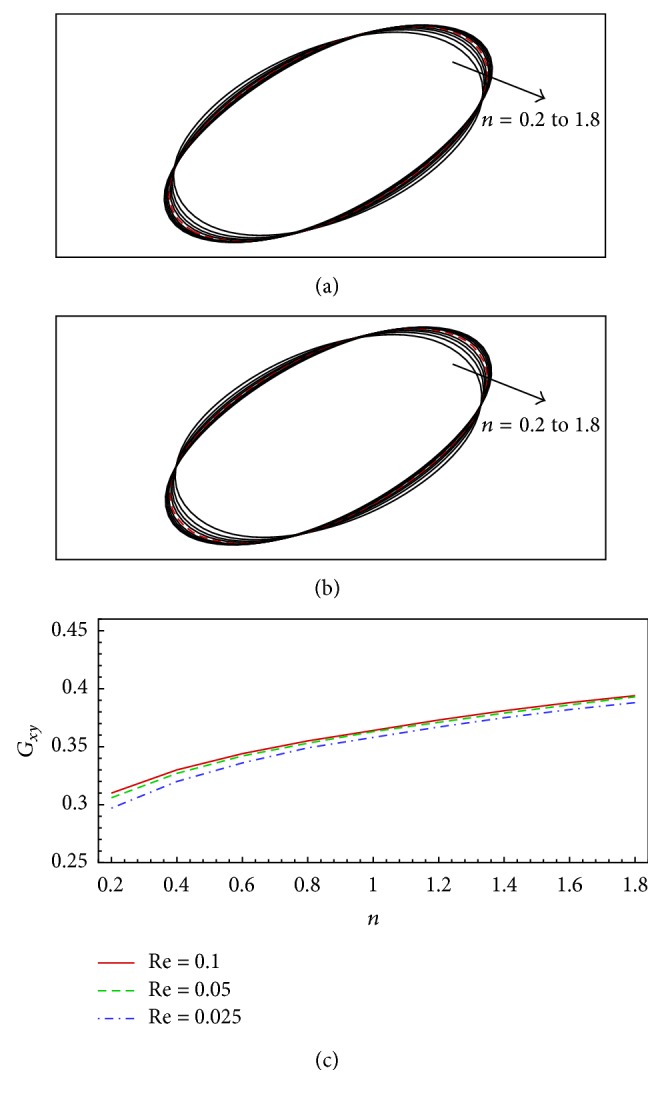
Deformation of the flexible capsule in a shear flow for Reynolds number of Re = 0.1 and 0.025, dimensionless shear rate of *G* = 0.04, and power-law index of *n* = 0.2 to 1.8: (a) capsule shapes for difference power-law indices at Re = 0.1 (the dashed line is for the Newtonian fluid case where *n* = 1.0), (b) capsule shapes for difference power-law indices at Re = 0.025, and (c) Taylor shape parameter as a function of power-law index. *G*_*xy*_ for Re = 0.05 is shown in (c) for comparison.

In order to study the Reynolds number effect on the deformation of the capsule, we simulate two additional Reynolds numbers, Re = 0.1 and 0.025.  Figure 7 shows the deformation of the flexible capsule in a shear flow for Reynolds number of Re = 0.1 and 0.025, dimensionless shear rate of *G* = 0.04, and power-law index of 0.2 ≤ *n* ≤ 1.8. It is found that the deformation (*D*_*xy*_) for Re = 0.1 is larger compared to the cases of Re = 0.05 and 0.025. However, the difference is quite small, implying that, in the low Reynolds number regime, for example, Re ≤ 0.1 in this work, the deformation of the capsule is not significantly affected by the Reynolds numbers used, as the inertial force is ignorable here, and the shear forces and capsule elastic forces are dominant. Therefore, the dimensionless shear rate (*G*) should significantly affect the deformation of the capsule, which will be further verified by the simulations shown below by varying *G*.

**Figure 8:**
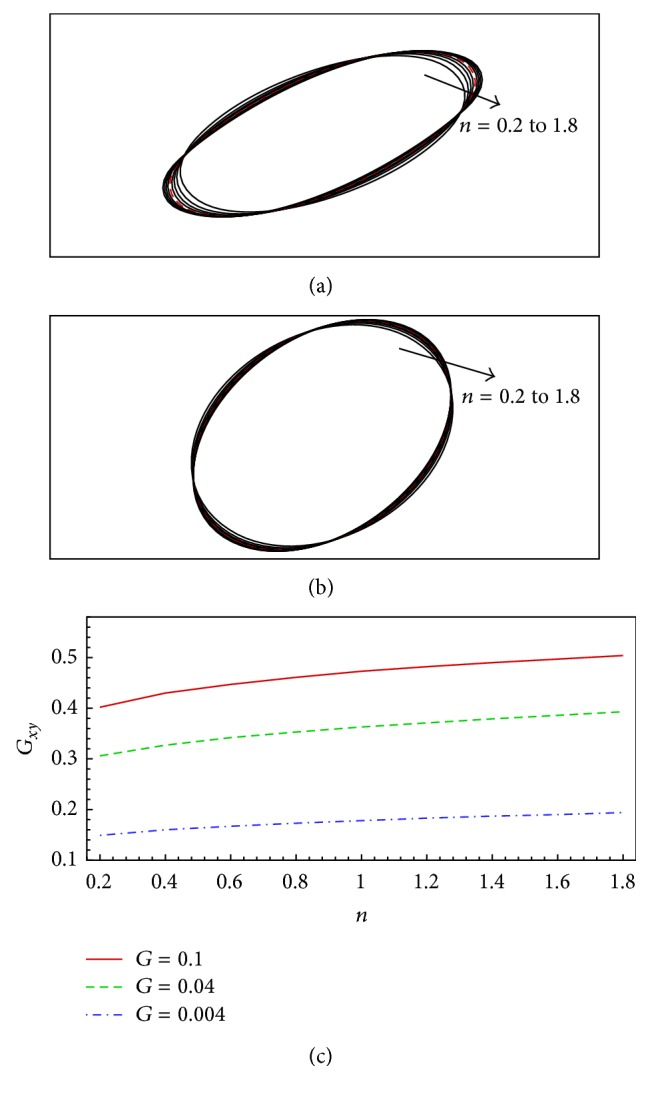
Deformation of the flexible capsule in a shear flow for dimensionless shear rate of *G* = 0.1 and 0.004, Reynolds number of Re = 0.05, and power-law index of *n* = 0.2 to 1.8: (a) capsule shapes for difference power-law indices at *G* = 0.1 (the dashed line is for the Newtonian fluid case where *n* = 1.0), (b) capsule shapes for difference power-law indices at *G* = 0.004, and (c) Taylor shape parameter as a function of power-law index. *G*_*xy*_ for *G* = 0.04 is shown in (c) for comparison.

Finally, we study the shear rate effect on the deformation of the capsule by using *G* = 0.004 and 0.1 at Re = 0.05. The deformation of the flexible capsule in a shear flow for dimensionless shear rate of *G* = 0.1 and 0.004, Reynolds number of Re = 0.05, and power-law index of *n* = 0.2 to 1.8 is shown in Figure 8, from which several interesting observations are obtained. First, the capsule deformation is sensitive to the dimensionless shear rate. This can be explained by the definition of *G* in (16): *G* measures the ratio of shear (viscous) forces to the stretching elastic forces, which is the dominant physical process here. A change of this ratio would cause significant difference in the capsule deformation. Second, the power-law index effect is stronger for larger *G*, as indicated by the slopes of the *G*_*xy*_ functions shown in Figure 8(c). This can be explained by the fact that the physical process changes from a shear force dominant to an elastic-force dominant process when *G* varies from 0.1 to 0.004. For low *G*, for example, 0.004, the elastic forces are dominant, and thus the shear force change caused by the change of the non-Newtonian rheology is smaller compared to that for larger *G*, for example, 0.1. Finally, the deformed capsule is obviously biased from elliptical cylinder for large *G* and *n*; for example, *G* = 0.1 and *n* ≥ 1.2. This is caused by the shear-induced torque on the deformed capsule and the decrease of effective bending resistance caused by the shear-induced elongation.

**Figure 9:**
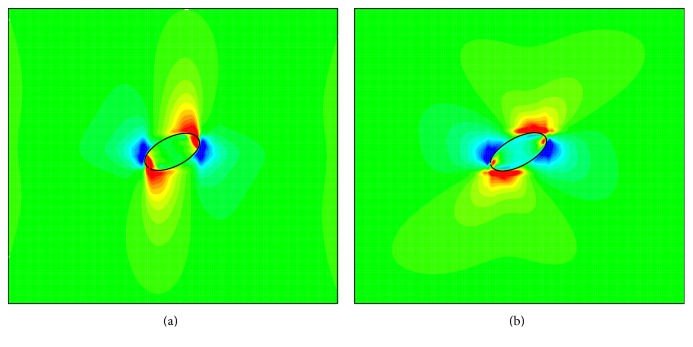
Contours of *χ* = −*μω* for *G* = 0.04 and Re = 0.025: (a) *n* = 0.6 and (b) *n* = 1.4.

To further discuss the non-Newtonian effect, *χ* = −*μω*, which measures the local shear stress, is introduced [40].  Figure 9 shows contours of *χ* for *G* = 0.04, Re = 0.025, *n* = 0.6, and 1.4. It finds that *χ* near the long axial ends is larger (i.e., the local shear stress is larger) for *n* = 1.4 compared to that for *n* = 0.6. This is a further explanation of larger deformation for larger *n*.

## 4. Conclusion

A numerical approach combining the immersed boundary method and the lattice Boltzmann method has been developed for fluid-structure interactions involving non-Newtonian fluids. Without loss of generality, the power-law fluid is taken as a representation of non-Newtonian fluids to present the method. This method couples the flexible structure (e.g., capsule) dynamics and the fluid dynamics by using the immersed boundary method and calculates the incompressible viscous power-law fluid motion by solving the lattice Boltzmann equation. In order to achieve the non-Newtonian rheology, a shear rate-dependant relaxation time is employed.

The non-Newtonian flow solver has been validated by conducting a power-law flow in a straight channel. The power-law index has been varied from 0.6 to 1.4. The present numerical results show a good agreement with the analytical solutions for various values of power-law index, giving us confidence in the reliability and accuracy of the present numerical solution procedure.

To study the non-Newtonian effects on the deformation of a capsule in a power-law shear flow, we have performed simulations by varying the Reynolds number from 0.025 to 0.1, dimensionless shear rate from 0.004 to 0.1, and power-law index from 0.2 to 1.8. It is found that the capsule deformation increases with the power-law index for different Reynolds numbers and nondimensional shear rates. In addition, the Reynolds number does not have significant effect on the capsule deformation in the flow regime considered. Finally, the power-law index effect is stronger for larger dimensionless shear rate compared to smaller values.
